# Medical Items

**Published:** 1916-05

**Authors:** 


### MEDICAL ITEMS

### RANGE EXTENSION OF CEANOTHUS SANGIUNEUS.

(Reprinted from Rhodora, Journal of the New England Botanical Club, Vol· 17---1915).

(Oliver A. Farwell.)

In the Synoptical Flora and in Piper's Flora of the State of Washington the distribution of Ceanothus sanguineus, Pursh is given as from Brit. Columbia to N. California and Idaho. Howell gives the same range but extends it eastward to Montant. Rydberg, in the Flora of Montana, says it occurs only west of the main range of the Rocky Mountains. Its discovery, Coulter's Manual, 1st. Ed., credits it to the region of the Missouri and its tributaries but it is not given a place in the 2nd. edition. Pursh, who first described it from material collected by Lewis, gives it as "Near the Rocky Mountains, on the banks of the Missouri"; in so far as this statement by itself is concerned, it might mean either the eastern or western slope of the Rockies or both; I have not access to any records that might determine the point in question. The plant, however, has been considered to belong exclusively to the northwestern region west of the main range of the Racky Mounatins. Its discovery, therefore, in the Keweenaw Peninsula, in Michigan, is of more than local interest. I first collected it in fruit in August, 1886, near Copper Harbor. At that time, being young in years and botanical experience, my main object was to make each species I found agree with some one of those enumerated in Gray's Manual; so it naturally found a resting place in the species cover of C. Americanus where it remained forgotten until 1914. Having in that year had occasion to examine critically my material of the eastern species I at once observed that the Keweenaw plant was not C. Americanus. An investigation convinced me that it was either C. sanguineus or a new species. On a trip to the Lake Superior region early in July of this year (1915) I found the shrub in full bloom. It proved to be C. sanquineus. I examined a section of territory about half a mile long by as much wide and found the plant to be quite plentiful; the probabilities are that it may be found over a much larger area. It is found in rocky woods, the rock being of trap rock formation; the woods are mostly of evergreen trees but have a good sprinkling of oak, birches, willows, poplars, maples and other deciduous trees and shrubs. It is, of course, indigenous to this region. The possibilities of its having been an introduction from west of the Rockies are so remote as to be negligible. Plants from the eastern slope of the Rockies might be introduced to the Lake Superior region by way of the rivers and streams which find an outlet through Lake Winnipeg, Winnipeg River, the Lake of the Woods, Rainy Lake River, and Pigeon River to Lake Superior but this line of travel would not transport the seed of a plant from the western slope, nor would the Missouri carry the seed from that region to Lake Superior. The same remarks apply to another plant, the Mimulus moschatus, Dougl., which is native to the same regions; it may have been introduced further east as an escape from cultivation but the argument will not apply here, since, so far as I have been able to learn, it was never cultivated in the Copper district of Michigan unless gathered for the purpose from the local native plant. The only probable explanation of such widely separated stations is that the species in preglacial times were more generally distributed but that the ice of the glacial period destroyed intermediate stations.

DEPARTMENT OF BOTANY, L.
PARKE, DAVIS & COMPANY,
Detroit, Michigan.

---

"Tongaline Tablets have given my wife quicker relief for rheumatism, with which she is frequently afflicted, than any other remedy. Tongaline Liquid is one of the principal ingredients in all my prescriptions for rheumatic and neuralgic complaints. Furthermore I find it very efficacious for a rheumatic condition which I acquire as the result of exposure from riding."

---

FOR THE PROTECTION OF THE PATIENT.

In this day of sophistication and substitution the earnest physician cannot be too careful in following up his prescriptions to see that his patients are given exactly what he wants them to have—and nothing else. Especially is this so in regard to remedies of exceptional quality, such as Gray's Glycerine Tonic Comp. Numerous imitations of this reliable tonic are constantly being foisted on the unsuspecting, and when as a consequence of the patient failing to get the original "Gray's," the expected results do not materialize the doctor's skill and ability are apt to be questioned. For the protection of his patient and in justice to himself, the physician should invariably write for "Gray's" as follows:

R. Gray's Glycerine Comp.
(Purdue Frederick Co.) One bottle—16oz.

By thus specifying an original package, the painstaking physician will safeguard his patients and insure his results.

### RENEWED CONFIDENCE IN THE BROMIDE TREATMENT OF EPILEPSY

In the management of epilepsy the bromides still continue to be the remedy par excellence. In treating many of these unfortunate patients, however, the relief experienced is purchased at the expense of gastric disturbance, unsightly skin eruptions such as acne, and many other discomforts.

Unfortunately many practitioners have felt that the use of a single salt—potassium bromide usually—was preferable in the treatment of epilepsy. As a consequence of this and the all too frequent administration of an impure product, much harm has been done and the usefulness of the bromide treatment has been repeatedly questioned.

With the extension of knowledge on this important subject, however, a change has taken place and our most successful clinicians have at last wakened to the fact that satisfactory results can be accomplished by the bromides in the management of epilepsy if absolutely pure salts are used, in suitable combination and over a sufficiently long period. Continued study and investigation have substantiated these conclusions beyond all possible question.

Peacock's Bromides—each fluid drachm of which contains fifteen grains of the combined pure and neutral bromides of potassium, sodium, ammonium, calcium and lithium—meet these requirements in every way and can be administered over long and protracted periods, not only with benefits of the most positive and gratifying character, but with surprising freedom from gastric irritation or "bromism." It can be said without question that Peacock's Bromides have given substantial aid in restoring faith and confidence in the bromide treatment of epilepsy by placing it on a practical and effective basis.

---

The increasing knowledge of endamebas and their relation to infections and other pathological conditions calls for an extension of ipecac therapy. The alkaloids of ipecac are specific for the endamebas and quickly destroy all motile forms but if given by mouth they cause great nausea. The hypodermatic injection of emetine is efficient but time-consuming, frequently painful and expensive.

Alcresta tablets of Ipecac, Lilly, have made it possible to administer the ipecac alkaloids by mouth in massive doses without causing vomiting or nausea. These tablets are uncoated and disintegrating but do not liberate the alkaloids until the compound reaches the alkaline intestinal juices. Thus vomiting and nausea are avoided. Both physicians and dentists are using these tablets with much success in dysentery and pyorrhea and also in many affections that are known to have their source in local foci of infection.

---

### THE PROSPECTIVE MOTHER

During pregnancy, as a result of the extra tax imposed on every organ, the prospective mother often approaches the critical period of parturition without the reserve vitality and strength she obviously needs. To build up her body and reinforce enfeebled or flagging functions is imperative, and here will be shown the great value of Gray's Glycerine Tonic Comp. Used throughout the later months of pregnancy and following confinement, it gives to the mother the stimulus and support so necessary to enable her to pass successfully through a trying period, and also to meet the still more exacting demands of lactation.

As a result, therefore, of the benefits derived from this dependable tonic and reconstructive, breast feeding is not only made possible, but so greatly facilitated that the family physician cannot fail to appreciate the aid it gives him in his effort to this end.

---

"I prescribe Tongaline very frequently as a remedy for excess of uric acid, which is often the cause of rheumatism, and it is my sheet anchor for that condition. I also find Tongaline very beneficial in muscular pains due to a sluggish liver and inactive bowels. When a patient comes to me complaining of soreness all over, I place him upon Tongaline and it has never disappointed me."

